# Genito Pelvic Pain/Penetration Disorder (GPPPD) in Spanish Women—Clinical Approach in Primary Health Care: Review and Meta-Analysis

**DOI:** 10.3390/jcm11092340

**Published:** 2022-04-22

**Authors:** María Berenguer-Soler, Antonio Navarro-Sánchez, Antonio Compañ-Rosique, Paloma Luri-Prieto, Ramón Navarro-Ortiz, Luis Gómez-Pérez, Carla Pérez-Tomás, Elsa Font-Juliá, Vicente F. Gil-Guillén, Ernesto Cortés-Castell, Felipe Navarro-Cremades, Angel L. Montejo, María del Ángel Arroyo-Sebastián, Virtudes Pérez-Jover

**Affiliations:** 1 Faculty of Psychology, Miguel Hernández University, 03202 Elche, Spain; mariia_269@hotmail.com (M.B.-S.); navarrosancheztony@gmail.com (A.N.-S.); v.perez@umh.es (V.P.-J.); 2 University Hospital of San Juan, 03550 San Juan de Alicante, Spain; af.company@umh.es (A.C.-R.); palomalurip@hotmail.es (P.L.-P.); carlotilla@yahoo.com (C.P.-T.); elsafontjulia@hotmail.com (E.F.-J.); 3 School of Medicine, Miguel Hernández University, 03550 San Juan de Alicante, Spain; luisgope@gmail.com (L.G.-P.); vte.gil@gmail.com (V.F.G.-G.); ernesto.cortes@umh.es (E.C.-C.); fenacr@gmail.com (F.N.-C.); 4 University Hospital of Torrevieja, 03186 Torrevieja, Spain; ray_hard_@hotmail.com; 5 General University Hospital of Elche, 03203 Elche, Spain; 6 Psychiatry Service, Clinical Hospital of the University of Salamanca, 37007 Salamanca, Spain; 7 Institute of Biomedical Research of Salamanca (IBSAL), Paseo San Vicente SN, 37007 Salamanca, Spain; 8 Nursing School, University of Salamanca, Av. Donantes de Sangre SN, 37007 Salamanca, Spain; 9 Primary Care Center of Muchamiel, 03110 Muchamiel, Spain; mdaarroyo@hotmail.com

**Keywords:** genito-pelvic/penetration pain disorder, dyspareunia, vaginismus, vulvodynia, sexual pain, women, female, Spain, Spanish scientific publications, primary health care

## Abstract

Sexuality is a component of great relevance in humans. Sexual disorders are a major public health problem representing a high prevalence in the general population. DSM-5 genito-pelvic pain/penetration disorder (GPPPD) includes dyspareunia and vaginismus (DSM-IV-TR). To assess the importance of research on these disorders in Spain, we evaluated the Spanish scientific publications of primary and community care. The objective was to quantify the magnitude of the publications of GPPPD in Spanish women in primary and community care. For this, we used the method of conducting a systematic review and meta-analysis of studies evaluating GPPPD. As main results, of the 551 items found, we selected 11 studies that met the inclusion criteria. In primary care in Spain, one in nine women has these disorders; the percentage of women with GPPPD in this study (raw data) was 11.23% (95% CI: 0–29%) (vaginismus 5%; penetration pain 8.33%; dyspareunia 16.45%). These percentages can differ of those from other countries, and they are at the top of the data of the European countries (9−11.9%). There is much variability in the studies found in the world with respect to the prevalence of these health problems.

## 1. Introduction

The sexuality of human beings includes biological, psychological, and socio-cultural aspects, and it is one of the key factors for wellbeing [1] throughout the entire life cycle of a person [2]. There is no normative standard for personal sexual behavior, and satisfactory sexual functioning is subjectively defined [1,2,3]. Both disease-oriented and patient-oriented sexuality approaches are particularly important [4].

General medical conditions and mental disorders can have negative effects on human sexuality, and medication used also can have adverse effects on sexuality [1,4,5,6,7,8].

Sexual disorders are highlighted in the International Classification of Diseases (ICD) [9] and the Diagnostic and Statistical Manual of Mental Disorders (DSM) [10,11,12,13]. The current DSM-5 [10] includes vaginismus and dyspareunia in the new category of genito-pelvic pain/penetration disorder (GPPPD). The previous DSM-IV-TR included dyspareunia and vaginismus as separate entities [11]. Dyspareunia is a persistent or recurrent pain with attempted or complete vaginal entry and/or vaginal sexual intercourse; vaginismus is a persistent or recurrent difficulties to allow vaginal entry of a penis/finger/any object despite the woman’s expressed wish to do so [2,11]. 

The ICD-10 [14] includes sexual disorders under the “mental disorders”. They are included in the new chapter, Conditions Related to Sexual Health (CRSH) in ICD-11 [3,9,15]. ICD-10 F52 includes vaginismus (F52.5) and dyspareunia (F52.6) [14]. Current ICD-11 includes a grouping of sexual pain disorders (SPD) [3,9,15]. The sexual pain penetration disorder (SPPD) ICD-11 [15] category (HA20) includes vaginismus, excluding dyspareunia and vulvodynia, which are classified in the genitourinary chapter [15]. 

Several authors consider sexual pain disorder(s) to be pain disorders that interfere with sexuality rather than a sexual dysfunction [16]. 

Female sexual pain disorders are widely prevalent in women of all populations, ages, and life stages. [16]. In primary care in Spain, the percentage of women with GPPPD in this study was (raw data) 11.23%. These percentages can differ of those from other countries, and they are at the top of the data of the European countries (9–11.9%). There is much variability in the studies found in the world with respect to the prevalence of these health problems.

The highly variable results in prevalence figures may be due, at least partially, to the use of different concepts, definitions, and diagnostic criteria that do not always coincide; the use of different research instruments; as well as to a real difference in prevalence, among other possible explanatory variables [3,16].

Justification and working hypothesis: In view of the difficulty in finding studies that could resolve certain existing gaps in information on this disorder, we carried out a systematic review and meta-analysis of the clinical epidemiological studies performed to in Spain to determine the prevalence of GPPPD, specifically penetration pain, dyspareunia, and vaginismus in primary health care female patients attending health centers of Spain or its support services units.

Significance of this study: Many other studies on GPPPD and related diagnoses have been published in languages other than English, which hinders their access to the international scientific and clinical community using English language, and the publicity of the studies carried out. For this reason, the publication of works, such as the current study in Spanish is pertinent to facilitate access and visibility of these epidemiological studies carried out in languages other than English.

Objective: To quantify the magnitude of GPPPD in women in the Spanish primary health care setting or its support services units.

## 2. Materials and Methods

### 2.1. Design

Systematic review of studies published in Spanish assessing GPPPD in primary health care in Spain.

### 2.2. Protocol

For practice guidance on reporting this systematic review, we have followed as far as possible the Cochrane Handbook for Systematic Reviews of Interventions version 6.3 (2022) [17], considered alongside the Preferred Reporting Items for Systematic reviews and Meta-Analyses (PRISMA) 2020 statement [18].

### 2.3. Selection Criteria

For the final analysis, we evaluated all the studies that met the two inclusion criteria and none of the exclusion criteria:

- Inclusion criteria: (1) All studies published in Spanish that aimed to analyze GPPPD in Spain and (2) studies including patients from primary health care centers or their support services in Spain.
- Exclusion criteria: (1) Editorials, letters to the editor, or clinical cases and (2) studies not undertaken in Spanish primary health care centers or their support services.

### 2.4. Information Sources and Search

Identification of primary studies was performed, with the final literature search completed on 1 May 2016. The search was restricted to the Spanish language. A clear question (PICO; Patient or problem, Intervention, Comparison intervention, Outcome) was identified [19] using the Medical Subject Heading terms and the Health Sciences Descriptors. Key words used were: genito-pelvic/penetration pain disorder, dyspareunia, vaginismus, vulvodynia, sexual pain, women, female, Spain, Spanish scientific publications, primary health care.

We searched the following databases using key words, controlled terms, and Boolean logic operators (OR, AND, NOT): PubMed, PubPsych, Dialnet, Psicothema, Medes, ISOC, Elsevier Scopus, and Google Scholar. The team searched gray literature, searching manually through Google for unpublished works in scientific journals and books.

### 2.5. Study Selection

The selection and methodological evaluation of the studies was performed independently by two reviewers. Discrepancies were resolved by discussion or consensus with a third reviewer.

First, titles and abstracts were assessed, and potential studies were selected. Second, the full-text articles were then evaluated to determine whether they met the selection criteria.

### 2.6. Data Extraction

For each of the articles eventually selected, the two reviewers extracted the following information using mainly the same procedure: author, year of publication, population, geographical area, clinical setting (primary care centers and its support centers), study design, sample size, diagnostic, classification system, and tools used.

### 2.7. Methodological Evaluation of the Studies

As these studies were observational, the methodological quality of the studies included in the review was evaluated using the STROBE Statement (Strengthening the Reporting of Observational studies in Epidemiology) report [20] in addition to Cochrane standards [17] and PRISMA statement [18].

The methodological quality of the identified relevant publications was assessed using the Joanna Briggs Institute (JBI) critical appraisal tool for systematic reviews [21]. The JBI critical appraisal checklist for analytical cross-sectional studies and for case-control studies [22] of 8 and 10 items, respectively, were used. Reviews that met 75% of the JBI checklist criteria were classified as high quality. Those that met 50% of the criteria were classified as admissible quality. Those below 50% were considered low quality, and they were excluded from the meta-analysis [23].

### 2.8. Statistical Analysis

A statistical analysis and pooling of the results obtained from each selected study was performed with the statistical program StatsDirect-3 [24].

An Excel spreadsheet was used to record the number of women with GPPPD during coital intercourse and the total number of patients evaluated from each of the studies. The number of women with GPPPD was calculated based on the percentage and total number of women evaluated. We calculated the overall percentage of women with GPPPD for each study with its 95% confidence interval (CI) and the weight of each study.

To compute the weighted mean, the fixed-effects model was used when there were no statistical differences in the homogeneity of the studies or the random-effects model when there were statistical differences [25,26].

To test the homogeneity of the studies, we used Cochran’s Q test [27] with a significance level of *p* < 0.01 and the I^2^ statistic [27,28] (percentage of variability of effect estimates that are due to heterogeneity. Values of 25%, 50%, and 75% in the I^2^ test correspond to low, medium, and high levels of heterogeneity, respectively).

In the fixed-effects model, differences among the studies were assumed to be due to chance. In the random-effects model, we hypothesized that the variation among the studies was not attributable solely to chance but that we had to consider other reasons for the occurrence of the heterogeneity.

When studies are homogeneous, the fixed- and random-effects methods yield similar results; however, when heterogeneity exists, the confidence interval will be wider with the random-effects method.

The results of the meta-analysis are presented in a forest plot [29,30] in which the proportion of women with GPPPD from each individual study is plotted with its confidence interval. The mean value for each study is represented by a square with an area proportional to the weight in the overall calculation. The lower part of the plot shows the weighted mean of the women with GPPPD.

### 2.9. Risk of Bias

The team assessed possible publication bias [31,32] with the Begg-Mazumdar [33], Harbord [34], and Egger [35] tests, with a significance level of *p* < 0.01. Publication bias was also assessed visually using a funnel plot [32]. This is a scatter plot illustrating the relationship between the effect size (*x*-axis) and the standard error (*y*-axis) of each study.

For the correct interpretation of the results of a test to detect publication bias, both the number of studies (minimum 10) and the presence of heterogeneity in the meta-analysis should be taken into account. An asymmetric plot suggests the existence of publication bias. However, it should be mentioned that funnel plots have a disadvantage: symmetry is subjectively defined by the researcher [32,36].

## 3. Results

According to the Cochrane standards [17] and the PRISMA statement [18] applied to observational studies and the STROBE report [20], all the studies were considered to be of acceptable methodological quality for inclusion in the pooled analysis.

**Figure 1:**
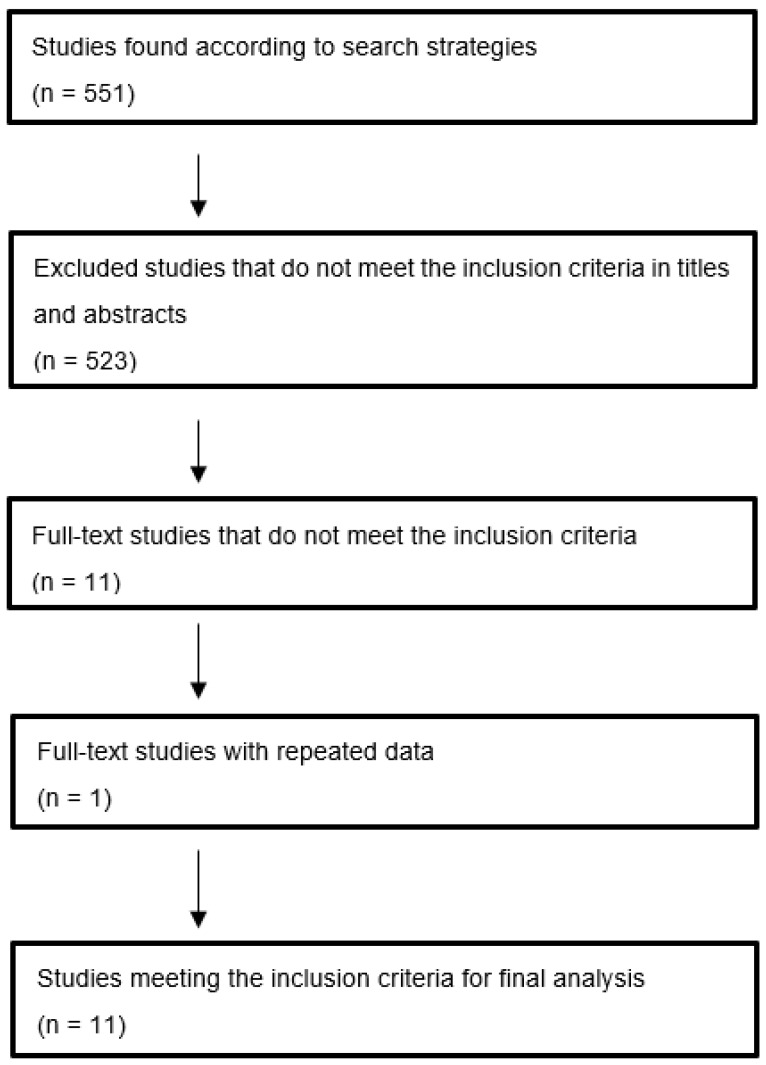
Flowchart of the literature search.

Flowchart of the literature search: The number of studies obtained from the databases was 551, of which 11 were selected for the final analysis (Figure 1).

Excluded studies that do not meet the inclusion criteria in titles and abstracts.

n = 523 studies. n = 5 doctoral theses.

Full-text studies that do not meet inclusion criteria. n = 11 studies.

Full-text studies with repeated data. n = 1.

Studies meeting inclusion criteria for final analysis. n = 11.

The 11 studies analyzed (Table 1) are listed by author and year, number of women evaluated, number of patients, and percentage of women with GPPPD.

None of the included publications met all the criteria of the JBI checklists. Five studies were rated as high quality and six as admissible quality. Supplementary Table S1 shows the quality assessment of included studies. No study was excluded from the meta-analysis.

Table 2 shows the results of the tests of publication bias, for which the results were not significant (Begg-Mazumdar *p* = 0.3587, Egger *p* = 0.1065, and Harbord *p* = 0.4142).

**Figure 2:**
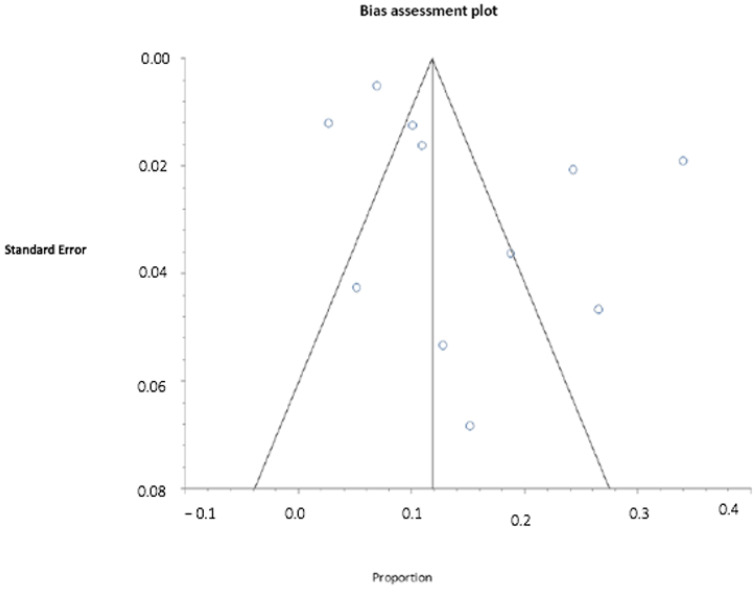
Funnel plot. The graphical equivalent of publication bias tests of Table 2. These small circle are the graphical representation of the final studies selected (n = 11).

The graphical equivalent of these publication bias tests (Figure 2): in the funnel plot [32], the proportion of women with genito-pelvic pain (measured effect) is represented on the *x*-axis and the standard error (measure of precision) on the *y*-axis. The absence of publication bias is determined by the symmetry of the point cloud, where each point represents a study.

**Figure 3:**
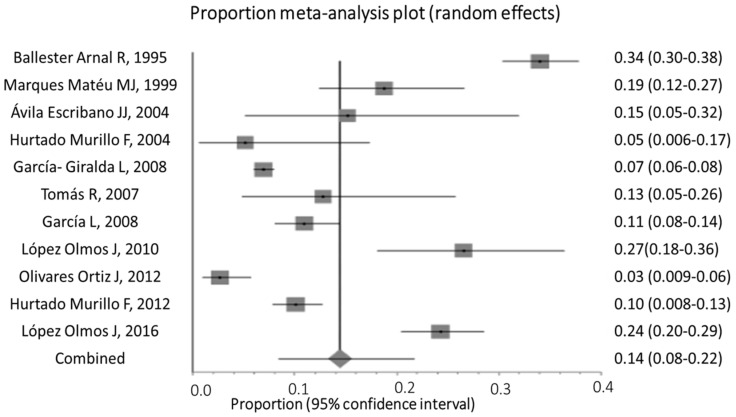
Forest plot of the pooled analysis for the random-effects model.

The forest plot [29,30] of the pooled analysis for the random-effects model is described graphically (Figure 3). Studies with larger samples correspond to narrower CIs.

In the final selected studies (n = 11), selected data (%, range, mean, and the number of studies (n)) for each diagnosis are presented in Supplementary Table S2.

Studies meeting inclusion criteria for final analysis (n = 11) are described in Table 3.

Forest plot of the pooled analysis for the random-effects model is shown in the Figure 3.

The presence of homogeneity was taken into account for the pooling of the results. Cochran’s Q value was 367.7 (degrees of freedom = 10); *p* < 0.0001, and the I^2^ statistic index was 97.3% (95%CI: 96.6–97.8%). The results indicated significant differences between the studies. Therefore, no homogeneity was considered to exist between the studies, and they were more different from each other than would be expected if it were due to random error alone. Accordingly, the random-effects model was used to compute the weighted mean.

Using the random-effects model, the weighted mean percentage of women with sexual pain was 14.43% (95%CI 8.44–21.70). The number of women evaluated was 5295, with an age range of 14 to 70 years, of whom 685 women had GPPPD.

## 4. Discussion

The analysis of the 11 selected studies [37,38,39,40,41,42,43,44,45,46,47] of sexual dysfunction published in Spain, using the random-effects method due to the high heterogeneity among the studies, revealed that one in nine women in the primary care setting suffers from genito-pelvic pain. GPPPD affects (raw data) to 11.23% of women, specified as vaginismus (5%), penetration pain (8.33%), and dyspareunia (16.45%).

The random-effects method assumes that in addition to intra-study variability, variability also exists among the different studies and that the sample of these studies is random from the entire population analyzed. In the past, an I^2^ value of less than 50% was considered acceptable to perform a meta-analysis. However, it is now acknowledged that even if there is statistical heterogeneity, a meta-analysis can be performed. In meta-analysis, we must consider clinical heterogeneity as the differences in participants, interventions, and results and methodological-statistical heterogeneity as the differences in study designs and measures of effects [48,49].

One of the causes of the heterogeneity of the studies was due to combining studies with different methodologies, populations, or quantification methods (sexual dysfunction questionnaires, clinical interviews, history checking). However, even when heterogeneity was present, pooling the results of the studies allowed us to gain information regarding the aim of this study [48,49].

Search bias was minimized by searching multiple databases (PubMed, PubPsych, Dialnet, ISOC, Medes, Psicothema, Elsevier Scopus, Google Scholar). In addition, a manual search was performed of the references of each study.

The publication bias found was minimal and not significant. The selected studies therefore adequately represent all the studies carried out on GPPPD in women in Spain. The funnel plot [32] was quite symmetrical, indicating that publication bias was minimal.

In the Global Study of Sexual Attitudes and Behaviors (GSSAB) [50] the percentage of women with “Pain during sex” in southern Europe, including Spain, was 11.9% (10.3, 13.4), and in northern Europe, this was 9% (7.5, 10.4), both below our percentage, which was 14.4%. In the rest of the countries, the figures ranged from 14% to 31.6% [50].

Estimating the prevalence of GPPPD can be complicated. GPPPD prevalence rates varied depending on the assessment used. When women were asked to self-report their experiences, one-third of them said they felt pain during intercourse or felt fearful of intercourse [51].

Results from an Iranian study [51] showed that 33% of women complained about pain during or fear of intercourse.

The results obtained by this study show that GPPPD is quite prevalent [51].

Cultural, religious, and other factors can affect results of differential manner [51].

Two highlighted surveys are the “Painful sex (dyspareunia) in women: prevalence and associated factors in a British population probability survey” using data of Natsal-3 (The Third National Survey of Sexual Attitudes and Lifestyles) from participants interviewed at home between 2010 and 2012 [52], and the GeSiD German survey, with data collected from October 2018 to September 2019 [53].

Painful sex (as dyspareunia) is a common but neglected female health problem. Its estimated prevalence in the population can vary from 3% to 18% globally, and its lifetime estimates range from 10 to 28%. Wide ranges of prevalence studies can reflect significant heterogeneity in main study factors, such as methodologies, sampling approaches, or/and other factors [52].

There is a strong link between GPPPD and impaired female sexual function [52,53] and with self-assessed poor health and experience of chronic health conditions [52].

Sexual pain prevalence using ICD-11 Guidelines (GeSiD German survey) [53] shows a lifetime prevalence of 20.6 (18.6, 22.9) and a 12-month prevalence of 10.9 (9.5, 12.4).

The Natsal-3 survey [52] was based on the criteria of the DSM-5. These expanding criteria of morbidity include some features of sexual dysfunction that affect estimates of prevalence, strongly lowering this: 22.8% of the women manifested at least one sexual problem, but signs of a disorder were only found in 3.6%; the prevalence of sexual dysfunction depends on age. Almost half of the women who cited sexual pain declared to be significantly impaired by it [52].

Prevalence estimates of sexual problems were quite similar in GeSiD [53] and Natsal-3 [52] surveys.

Future research should take into account the following proposals: To investigate possible sources of heterogeneity in the studies, determine their influence on the results, and to evaluate the possibility of performing subgroup analyzes. Multicenter studies should use the same methodology and criteria using accurate standardized diagnostics DSM5 and/or ICD-11. Cross-sectional studies should be continued with prospective follow-up. The use in the clinical practice of psychometric instruments should be validated and cross-culturally adapted as well as standardized and referring to the diagnostic criteria DSM-5 and/or ICD-11.

Future studies should take into account the best scientific evidence available and applicable to clinical practice in real conditions with decision making by the clinician centered on the patient, according to the usefulness and applicability of this evidence to each specific patient and their preference of this and always in benefit of it.

Limitations. The present study is limited to the Spanish population and clinical settings, that is, women treated in primary health care or its reference services in Spain; consequently, the generalizability of our results to the whole population of women with GPPPD is limited. It is restricted to publications of studies conducted in Spain, in the Spanish language, published in Spanish scientific journals, without including or considering works published in English and outside of Spain. The studies selected in this systematic review are observational with significant heterogeneity. Due to the limited number of final publications selected of the study (n = 11), its findings should be interpreted with caution; more studies are needed to obtain more consistent conclusions on this topic.

Future directions. Research should take into account the following proposals: To investigate possible sources of heterogeneity in the studies, determine their influence on the results, and to evaluate the possibility of subgroup analysis within each study. Multicenter studies should use the same methodology and criteria from standardized diagnostics DSM5 and/or ICD-11. Cross-sectional studies could be continued along with prospective studies. The use in the clinical practice of psychometric instruments should be validated and cross-culturally adapted as well as standardized and referring to the diagnostic criteria DSM-5 and/or ICD-11.

## 5. Clinical Approach

Sexuality, when is perceived as self-satisfying, has a main positive impact on quality of life (QoL) [54]. Physical and mental illnesses can decrease QoL and have a significant negative effect on sexual activity and sexual satisfaction [54]. The evidence of the relationship between sexuality and health robustly contributes to its current expanding awareness in the psychological and medical settings both clinical and research, connecting people’s mental and physical health with individual sexual activity and sexual satisfaction [54]. However, sexuality and specifically sexual dysfunction still play a limited role in daily clinical practice in patients of all age groups [54,55].

Both sexual disorders and sexual problems are especially prevalent among general medical and psychiatric patients, including medical treatments [5,6,7,8]. DSM and ICD models have distinctive constituents and purposes [3,9]. DSM-5 includes only mental disorders. ICD-11 (WHO) covers all health conditions for the generality of countries [3,9].

Human sexual response is usually experienced in a broad context, including intrapersonal, interpersonal, and cultural [56]. Sexual function includes a complex interaction between biological, psychological, and sociocultural factors [56].

It is important to make the distinction between sexual disorders described in the current classifications (DSM and ICD systems) [2,3,9] and the sexual problems and difficulties in daily life [56], which are transient alterations or interruptions in sexual functioning [56].

DSM-5 criteria provide a descriptive system in building the diagnosis of female sexual dysfunction as well as its etiology [56], along with the information that can be obtained from a comprehensive clinical interview and physical examination as well as complementary medical testing [56].

The evaluation of sexual dysfunction with DSM-5 criteria takes into account a series of associated characteristics to be considered since they may be relevant to clarify the etiology and/or for treatment [56]. DSM-5 includes the factors related to: individual vulnerability factors, psychiatric comorbidity, or stressors; partner; relationship; cultural or religious factors; and medical factors relevant to prognosis, course, or treatment. [10,56]. All these factors should be taken into account by clinicians in their everyday clinical practice.

Clinical differences between dyspareunia and vaginismus can be defined by the former DSM-IV-TR criterion A. Criterion A (symptomatology) of dyspareunia is the “recurrent or persistent genital pain associated with sexual intercourse in either male or female” [11]. Criterion A of vaginismus is the “recurrent or persistent involuntary spasm of the musculature of the outer third of the vagina that interferes with sexual intercourse” [11].

Dyspareunia and vaginismus in DSM and ICD classifications are summarized in Supplementary Table S3.

Healthcare professionals may be uncomfortable addressing the patient’s sexuality aspects in the clinical interview for various reasons. Nevertheless, the evaluation of patients sexual functioning should be part of all comprehensive evaluation by every healthcare professional [56].

To establish the diagnosis of GPPPD, the beginning is the clinical interview followed by the physical examination, including a pelvic examination [56], and the performing of complementary studies to determine any organic factors underlying the diagnosis of GPPPD [56,57]. Biological causal and related factors include different illness and associated treatments, including surgical procedures, such as thyroid autoimmune disease [58], endometriosis [59,60,61,62], medical treatments [61], surgical treatment techniques [60,63,64], hidradenitis suppurativa [65], localization of pain sensitivity in insertional dyspareunia [66], and chronic pelvic pain [67]. The collaboration of sexual medicine experts from scientific societies with the ICD-11 provides confidence on its codes for diagnosing and coding sexual dysfunctions, including sexual pain disorders, in worldwide clinical settings and providing sexual medicine experts with a greater understanding of risk factors and etiologies of sexual pain disorders [68].

For the use of psychometric instruments, there is a serious shortage of standardized, transculturally adapted and validated instruments can be used to assess GPPPD in women [56]. Some self-report questionnaires include SPD, SPPD, GPPPD, dyspareunia, and vaginismus [56,57].

## 6. Conclusions

In the field of primary care in Spain, 11.56% women present GPPPD. In addition, the selected studies present an acceptable methodological quality for meta-analysis. There is considerable variability in the data on the prevalence of GPPPD among the included studies. However, this variability may be explained in an large part by the different measurement instruments used since homogeneity in the methods is desirable. The prevalence of a given sexual disorder will depend to a large extent on the definition of the disorder, the population studied, and the methodology used.

## Figures and Tables

**Table 1:** Studies of GPPPD.

Author/Year	No. of Women Evaluated	Study Location
Ballester Arnal R, 1995 [37]	644	Population, Valencia-Castellón
Marques Matéu MJ, 1999 [38]	128	SRHM *, Villareal
Ávila Escribano JJ, 2004 [39]	33	Alcoholism Unit, Salamanca
Hurtado Murillo F, 2004 [40]	39	SRHM *, Valencia
Tomás R, 2007 [41]	47	Health center, Albacete
García-Giralda L, 2008 [42]	2599	Health centers in Spain
García L, 2008 [43]	403	Health center, Murcia
López Olmos J, 2010 [44]	98	Specialty center, Valencia
Olivares Ortiz J, 2012 [45]	226	Health center, Madrid
Hurtado Murillo F, 2012 [46]	625	SRHM *, Valencia
López Olmos J, 2016 [47]	453	Specialty center, Valencia

* SRHM (Sexual and Reproductive Health Medicine) center; author/year; no. of women evaluated; Study location: population and setting as SRHM, alcoholism unit, health centers (primary care), specialty centers.

**Table 2:** Tests of publication bias.

Begg-Mazumdar: Kendall’s tau = 0.236364 *p* = 0.3587
Egger: bias = 4.057145 (95% CI = 1.060592 to 9.174882) *p* = 0.1065
Harbord: bias = 3.144143 (92.5% CI = 4.248425 to 10.536711) *p* = 0.4142

**Table 3:** Studies (n = 11) meeting inclusion criteria of final analysis. Descriptive data, GPPPD specifically referred as penetration pain, dyspareunia, vaginismus; expressed as a percentage.

Authors	Type of Study/Design	Intervention	Results
Ballester Arnal R, 1995	Analytical cross-sectional study	BES questionnaire not validated. 20 items. Two main variables: experience of suffering from sexual dysfunctions (DSM III-R) and item about the size of the penis. DSM-III-TR	Dyspareunia 29% Vaginismus 5%
Marques Matéu MJ, 1999	Case control study	Reference to DSM-IV.	Dyspareunia19.5% Vaginismus 5%
Ávila Escribano JJ, 2004	Analytical cross-sectional study	Self-administered and anonymous survey containing sociodemographic questions and the LoPiccolo Sexual History Questionnaire	Dyspareunia 5% Vaginismus 10%
Hurtado Murillo F, 2004	Analytical cross-sectional study	Self-Applied Marital Adjustment Scale; Beck-depression inventory BDI, State Trait Anxiety questionnaire, Personality Questionnaire EPI, Seef- esteem survey EAE	Dyspareunia 5.13% Vaginismus 0%
Tomás R, 2007	Analytical cross-sectional study	Psychological well-being (IBP questionnaire) and quality and satisfaction (LISAT-8). A questionnaire was developed following DSM-IV criteria	Penetration Pain 12.8%
García-Giralda L, 2008	Analytical Cross-sectional, multicenter study	Validated questionnaire on female sexual health and dysfunction (SyDSF). DSM-IV-TR.	Penetration Pain 6.93%
García L, 2008	Analytical cross sectional multicenter study	Validated questionnaire on female sexual health and dysfunction (SyDSF). DSM-IV-TR.	Penetration Pain 11%
López Olmos J, 2010	Case-control study	Female health and sexual dysfunction questionnaire in primary care (SyDSF-Ap)	Dyspareunia 26.92%
Olivares Ortiz J, 2012	Analytical cross-sectional study	FMS questionnaire for female sexual disfunction	Penetration Pain 2.6%
Hurtado Murillo F, 2012	Analytical cross-sectional study, clinical sample; SD	Review of medical records	Dyspareunia 5.3%
López Olmos J, 2016	Analytical cross-sectional study	Brief Profile of Female Sexual Function (BPFSF) questionnaire.	Dyspareunia 24.28%

## Data Availability

Data is contained within the article.

## Supplementary Materials

The following supporting information can be downloaded at: https://www.mdpi.com/article/10.3390/jcm11092340/s1, Table S1: The quality assessment of included studies. Cross-sectional studies (nine) and Case-control studies (two); Table S2: Studies (n = 11) meeting inclusion criteria of final analysis. For each diagnosis, its %, range, mean and the number of studies (n) are presented; Table S3: Dyspareunia and vaginismus selected items in the classifications DSM-IV-TR, DSM-5, ICD-10 and ICD-11.
