# Supplementary material for: Genito Pelvic Pain/Penetration Disorder (GPPPD) in Spanish Women—Clinical Approach in Primary Health Care: Review and Meta-Analysis
Source: J Clin Med. 2022 Apr 22;11(9):2340. doi: 10.3390/jcm11092340 (PMC9105657; doi:10.3390/jcm11092340)
Supplement: Supplementary file 1 [file jcm-11-02340-s001.zip › jcm-1659211-supplementary.pdf]

Supplementary Table S1. The quality assessment of included studies. Cross-sectional studies (nine) and Case-control studies (two).

	Items	1	2	3	4	5	6	7	8	TOTAL
Cross sectional studies	Ávila Escribano JJ-2004	✓	✓	X	✓	✓	X	✓	✓	6/8
	Ballester Arnal R-1995	X	✓	X	✓	✓	X	X	✓	4/8
	García-Giralda Ruiz, L-2008	✓	✓	✓	✓	X	X	✓	?	5/8
	Estudio VASS.AP-2008	✓	✓	✓	✓	X	X	✓	?	5/8
	Hurtado Murillo F-2004	✓	✓	✓	?	X	X	✓	✓	5/8
	Hurtado Murillo F-2012	✓	✓	✓	✓	✓	X	✓	✓	7/8
	Olivares Ortiz J-2012	✓	✓	✓	✓	✓	X	✓	✓	7/8
	Tomás R-2007	✓	✓	X	✓	X	X	✓	✓	5/8
	López-Olmos J-2010	✓	✓	✓	✓	X	X	✓	✓	6/8

	Items	1	2	3	4	5	6	7	8	9	10	TOTAL
Case-Control studies	López-Olmos J-2010	?	✓	✓	✓	✓	X	X	X	✓	✓	6/10
	MarquesMatéu MJ-1999	✓	✓	✓	✓	✓	X	X	✓	✓	✓	8/10

Supplementary Table S2. Studies (n = 11) meeting inclusion criteria of final analysis. For each diagnosis, its %, range, mean and the number of studies (n) are presented.

Diagnostic [Cites]	Range	Mean	Results, Raw Data, (and Number of Studies)
Vaginismus [37–40]	0–10%	5%	(%) 0; 5; 5; 10 (n = 4)
Dyspareunia [37–40,44,46,47]	5–29%	16.45%	(%) 5; 5,13; 5,3; 19,5; 24,28 *; 26,92; 29 (n = 7)
Penetration Pain [41–45]	2.6–12.8%	8.33%	(%) 2.6; 6.93; 11; 12.8 (n = 4)

* Premenopausal subgroup, 15,72% (n = 229); Postmenopausal subgroup, 33,03% (n = 224) [47].

Supplementary Table S3. Dyspareunia and vaginismus selected items in the classifications DSM-IV-TR, DSM-5, ICD-10 and ICD-11.

DSM (APA)/ICD (WHO) Classifications: DSM Includes only Mental Disorders; ICD Covers All Health Conditions	Dyspareunia (D)	Vaginismus (V)
DSM-IV-TR [11], D,V, as separate entities	"Recurrent or persistent genital pain associated with sexual intercourse in either male or female" (Criterion A)	"Recurrent or persistent involuntary spasm of the musculature of the outer third of the vagina that interferes with sexual intercourse" (Criterion A)
DSM-5 [10] merged both D,V in a single diagnostic, Genito-pelvic pain/penetration disorder (GPPPD)	Genito-pelvic pain/penetration disorder (GPPPD)	Genito-pelvic pain/penetration disorder (GPPPD)
ICD-10 [14] mental and behavioral disorders chapter F52. Sexual dysfunctions, not caused by organic disorder or disease. D,V, as separate entities	In mental and behavioral disorders. (F52-6) Nonorganic dyapareunia. Pain during sexual intercourse. Excludes: dyspareunia (organic) (N94.1)	In mental and behavioral disorders. (F52-5) Nonorganic vaginismus. "Spasm of the pelvic floor muscles that surround the vagina, causing occlusion of the vaginal opening. Penile entry is either impossible or painful". Excludes: vaginismus (organic) (N94.2)

ICD-11 [15] D,V, as separate entities. Not classified in mental and behavioral disorders	Classified in the Diseases of the genitourinary system chapter. Sexual pain disorders grouping, inclusions: Psychogenic dyspareunia; a "Condition related to sexual health" (CRSH).	Sexual pain-penetration disorder within grouping Sexual pain disorders. A "Condition related to sexual health" (CRSH)
